# Monte Carlo Simulation of Errors for N-localizer Systems in Stereotactic Neurosurgery: Novel Proposals for Improvements

**DOI:** 10.7759/cureus.13393

**Published:** 2021-02-17

**Authors:** Mark Sedrak, Armando L Alaminos-Bouza, Andres Bruna, Russell A Brown

**Affiliations:** 1 Neurosurgery, Kaiser Permanente, Redwood City, USA; 2 Medical Physics, Mevis Informática Médica Ltda, São Paulo, BRA; 3 Director, Fi.Me. Fïsica Médica Srl, Córdoba, ARG; 4 Principal Engineer, Retired, Palo Alto, USA

**Keywords:** stereotactic frame, deep brain stimulation, n-localizer

## Abstract

Introduction: Frame-based stereotaxis has been widely utilized for precise neurosurgical procedures throughout the world for nearly 40 years. The N-localizer is an integral component of most of the extant systems. Analysis of targeting errors related to the N-localizer has not been carried out in sufficient detail. We highlight these potential errors and develop methods to reduce them.

Methods: N-localizer systems comprising three and four N-localizers of various geometries were analyzed using Monte Carlo (MC) simulations. The simulations included native and altered geometric dimensions (Width [W] x Height [H]). Errors were computed using the MC simulations that included the x- and y-axes of vertically oriented rods, that altered the W/H ratio, and that added a fourth N-localizer to a three N-localizer system.

Results: The inclusion of an overdetermined system of equations and the geometries of the N-localizer systems had significant effects on target errors. Root Mean Square Errors (RMS-e) computed via millions of MC iterations for each study demonstrated that errors were reduced by (1) inclusion of the x- and y-coordinates of the vertically oriented rods, (2) a greater triangular area enclosed by the diagonal fiducials of the N-localizer system (stereotactic triangle), (3) a larger W/H ratio, and (4) an N-localizer system that comprised four N-localizers.

Conclusion: Monte Carlo simulations of Root Mean Square error (RMS-e) is a useful technique to understand targeting while using N-localizer systems in stereotactic neurosurgery. The application of vertical rod positions enhances computational accuracy and can be performed on any N-localizer system. Keeping the target point within the stereotactic triangle enclosed by the diagonal rods can also reduce errors. Additional optimizations of N-localizer geometry may also reduce potential targeting errors. Further analysis is needed to confirm these findings which may have clinical importance.

## Introduction

Frame-based stereotaxis has revolutionized precise neurosurgical procedures throughout the world. Stereotactic space localization is a key step in the use of frame-based systems that provides coordinates for direct targeting in the brain. For this localization step, the N-localizer has been the most commonly used system for the past 40 years [1-3]. Depending on the manufacturer, the N-localizer has several dimensions (i.e., width and height) and configurations. Therefore, a careful and robust analysis of these systems may suggest improvements to the accuracy of the localization step, which may directly impact the targeting during the neurosurgical procedure. 

In this study, we utilized Monte Carlo (MC) simulations to highlight potential targeting errors during the localization step by computing targets within the stereotactic surgical volume. We incorporated a mathematical optimization of overdetermined equations that exploits the existing geometry of N-localizer systems. Moreover, we analyzed various existing and theoretical N-localizer geometries. 

## Technical report

Mathematics of the stereotactic localization

The mathematics of three N-Localizers used together for stereotactic neurosurgery have been published and extensively used [1-5]. As a standard approach, the method computes three three-dimensional (3D) points along the diagonal rods, given three corresponding two-dimensional (2D) points of intersection of the diagonal rods with an axial computed tomography (CT) image. Then a transformation matrix can be constructed to transform from 2D to 3D and from 3D to 2D. This classic method operates under the predicate that any stereotactic image should include all necessary and sufficient information for registration to its corresponding stereotactic system in order to accurately compute the target. Typically, this method applies a determined system of equations that includes three equations per axis, which is the minimum number of required equations to compute three unknowns. 

Rather than utilizing a determined set of equations to compute the transformation matrix, the utilization of an overdetermined system of equations may minimize errors. The vertical fiducials represent a source of data that positions that may improve the computation and are not utilized directly in the determined system. In order to develop an overdetermined system of equations, we first need to set up a general transformation equation. The first step is to construct column vectors that convert 2D coordinates (u, v, t) to 3D coordinates (x, y, z) (Equations 1-3). In this transformation for imaging, the coordinate $t$ is generally set to any non-zero value such as 1 [1]. Then these equations can be assimilated into a general transformation matrix that easily accommodates an overdetermined system of equations (Equation 4). This overdetermined system of equations takes the optimized form that is solved by using a pseudoinverse technique that minimizes errors, such as with Singular Value Decomposition (SVD) or QR Decomposition [6].

$$\begin{bmatrix} u_{1} & v_{1} & t \end{bmatrix} \cdot \begin{bmatrix} m_{11} \\ m_{21} \\ m_{31} \end{bmatrix} = x_{1} \tag{1}$$

$$\begin{bmatrix} u_{1} & v_{1} & t \end{bmatrix} \cdot \begin{bmatrix} m_{12} \\ m_{22} \\ m_{32} \end{bmatrix} = y_{1} \tag{2}$$

$$\begin{bmatrix} u_{1} & v_{1} & t \end{bmatrix} \cdot \begin{bmatrix} m_{13} \\ m_{23} \\ m_{33} \end{bmatrix} = z_{1} \tag{3}$$

$$\begin{bmatrix} u_{1} & v_{1} & t & 0 & 0 & 0 & 0 & 0 & 0 \\ 0 & 0 & 0 & u_{1} & v_{1} & t & 0 & 0 & 0\\ 0 & 0 & 0 & 0 & 0 & 0 & u_{1} & v_{1} & t \\ \vdots & & & & & & & & \vdots\\ u_{n} & v_{n} & t & 0 & 0 & 0 & 0 & 0 & 0 \\ 0 & 0 & 0 & u_{n} & v_{n} & t & 0 & 0 & 0\\ 0 & 0 & 0 & 0 & 0 & 0 & u_{n} & v_{n} & t \end{bmatrix} \cdot \begin{bmatrix} m_{11}\\ m_{21}\\ m_{31} \\ m_{12}\\ m_{22}\\ m_{32} \\m_{13}\\ m_{23}\\ m_{33} \end{bmatrix} = \begin{bmatrix} x_{1}\\ y_{1}\\ z_{1} \\ \vdots\\ x_{n}\\ y_{n} \\ z_{n} \end{bmatrix}\tag{4}$$

The transformation matrix can then be rearranged into a 3x3 stereotactic transformation matrix (${ m }$) (Equation 5) that can then be used to transform 2D to 3D coordinates (Equation 6) or to transform 3D to 2D coordinates, where $m^{-1 }$ denotes the inverse of $m{ }$ (Equation 7). The determinant of $m{ }$ should be computed prior to computing the inverse and rarely can be zero at a perfectly orthogonal zero plane. It should also be noted that the inverse of $m{ }$ can also be computed by interchanging the (u, v, t) coordinates with the (x, y, z) in Equation 4 to avoid performing a matrix inverse operation. 

$$m = \begin{bmatrix} m_{11} & m_{12} & m_{13} \\ m_{21} & m_{22} & m_{23}\\ m_{31} & m_{32} & m_{33} \end{bmatrix} \tag{5}$$

$$\begin{bmatrix} u_{1} & v_{1} & t \end{bmatrix} \cdot \begin{bmatrix} m_{11} & m_{12} & m_{13} \\ m_{21} & m_{22} & m_{23}\\ m_{31} & m_{32} & m_{33} \end{bmatrix} = \begin{bmatrix} x_{1} & y_{1} & z_{1} \end{bmatrix} \tag{6}$$

$$\begin{bmatrix} u_{1} & v_{1} & t \end{bmatrix} = \begin{bmatrix} x_{1} & y_{1} & z_{1} \end{bmatrix} \cdot \begin{bmatrix} m_{11} & m_{12} & m_{13} \\ m_{21} & m_{22} & m_{23}\\ m_{31} & m_{32} & m_{33} \end{bmatrix}^{-1} \tag{7}$$

Monte Carlo simulations for error analysis

Monte Carlo (MC) simulations were performed to introduce random errors into the computation of the stereotactic transformation matrix (${ m }$). This process utilized 35x1024x1024 repeated random errors up to 1 millimeter (mm) per study set at 0.5 mm/pixel, which were isotropic in horizontal and vertical pixel widths. The utilization of MC simulations has been previously published for single localizer comparisons [4], but here we utilize MC simulations for the computation of the stereotactic transformation matrix (${ m' }$), which is defined on the left-hand side of Equation 4, using three or more localizers. MC simulation computes the maximum deviation of all cases. The ideal transformation matrix (${ m }$) and the perturbed MC computed transformation matrix (${ m' }$) were used with Equation 6 to compute a 3D target position, (${ P_{i} }$) and (${ P'_{i} }$), respectively. Finally, the Root Mean Square Error (RMS-e) for ${ n}$ target positions was computed as Equation 8. 

$$RMS {-e} = \sqrt { \frac{1}{ n} \sum_{i}^{n} (P_{i} - P'_{i})^2 } \tag{8}$$

Overdetermined system of equations to compute the stereotactic transformation matrix

**Figure 1:**
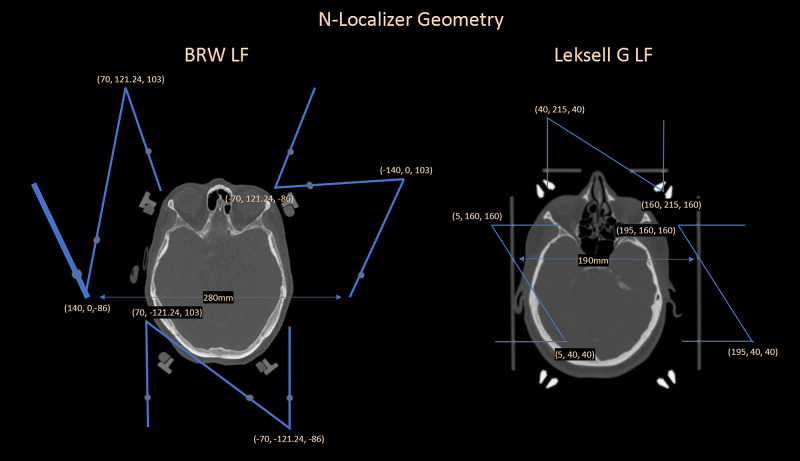
Geometry of the Brown-Roberts-Wells (BRW) localizer frame (LF) (left) and the Leksell G (LG) LF (right). The largest lateral diameter of the BRW LF spans a diameter of 280 mm and is larger than the overall geometry of the LG LF that measures 190 mm side-to-side. The BRW LF arranges the N-localizers circularly, whereas the LG LF arranges the N-localizers rectilinearly. Other frames, such as the Micromar and Fi.Me. frames, arrange the N-localizers rectilinearly as well but have slightly different overall geometry. The endpoint (x, y, z) coordinates of the N-localizers are displayed in the respective coordinate systems of the BRW LF and LG LF, depicted here in radiological convention. BRW, Radionics CRW Stereotactic System, Integra LifeSciences Corporation, Plainsboro, New Jersey Leksell G, Leksell Stereotactic Frame, Elekta, Stockholm, Sweden Micromar Ind. Com. Ltda, Diadema, Sao Paulo, Brazil Fi.Me. - Fisica Medica s.r.l, Cordoba, Argentina

In the analysis of Equation 4, an overdetermined system of equations can be used to compute the stereotactic transformation matrix (${ m }$). Then, analyzing the N-localizer system, one can identify several points via the vertical rods whose x-(lateral or LAT axis) and y-(antero-posterior or AP axis) coordinates are established by the manufactured geometry. Therefore, these vertical rod positions can be utilized in the same matrix computation for ${ m }$ to minimize errors related to the x- and y-axes. Some localizer systems also contain a fixed x- or y-axis along the diagonal rods, but this geometry is not universal and was not utilized in the analyses. We analyzed the localizer frames (LF) of two stereotactic systems: (1) the Brown-Roberts-Wells (BRW) LF (Radionics CRW Stereotactic System, Integra LifeSciences Corporation, Plainsboro, New Jersey, USA) and (2) the Leksell G (LG) LF (Leksell Stereotactic Frame, Elekta, Stockholm, Sweden), both depicted in Figure 1. The vertical rods lie at fixed locations relative to the x-axis and y-axis. These fixed locations are essential to computing the leftmost two column vectors of the transformation matrix (${ m }$), as shown in Equations 1 and 2, and to including these column vectors in the computation of the stereotactic transformation matrix (${ m '}$), as shown in Equation 4. 

The Root Mean Square error (RMS-e) for the average target positions within 50 mm from the center of the volume in each direction (center, lateral, anterior, and posterior) is shown in Table 1 for four different N-localizer configurations having various geometries, where each configuration comprises three N-localizers. RMS-e is clearly reduced in every configuration by the inclusion of more data to specify an overdetermined system of equations. For three rods, the equations are determined as the standard approach, whereas the use of six (three diagonals plus three verticals) and nine rods (three diagonals plus six vertical) creates an overdetermined system of equations for computing the lateral (x-axis) and anteroposterior (y-axis) components of the transformation matrix (${ m }$), as in Equations 1 and 2. The overdetermined system does not affect the vertical (z-axis), which is determined by three points. 

**Table 1:** Four different localizer frames systems are studied using Monte Carlo (MC) simulation via the introduction of random errors into the N-localizer fiducials used to compute the transformation matrix (${ m }$) followed by computation of targets within 50 mm of the center of the stereotactic volume. Root Mean Square errors (RMS-e) are computed for determined (three rods) and overdetermined (six and nine rods) systems of equations. The overdetermined systems of equations make use of the spatial correspondence between the (u, v) and (x, y) coordinates of the fixed vertical rods within each N-localizer. Inclusion of more rods via an overdetermined system provides a substantial improvement in the RMS-e. This improvement is related to the overdetermination of the anteroposterior (y) and lateral (x) vector components of the transformation matrix (${ m }$) by inclusion of the vertical rods. The precision of the vertical (z) coordinates was not improved by applying this method, considering that only three vertical (z) coordinates are available for the three unknowns corresponding to the vertical component in the transformation matrix (${ m }$). Millimeters (mm) Root Mean Square error (RMS-e) BRW, Brown-Roberts-Wells, Radionics CRW Stereotactic System, Integra LifeSciences Corporation, Plainsboro, New Jersey, USA Leksell G, Leksell Stereotactic Frame, Elekta, Stockholm, Sweden Micromar Ind. Com. Ltda, Diadema, Sao Paulo, Brazil Fi.Me. - Fisica Medica s.r.l, Cordoba, Argentina

Localizer Frame	Width (mm)	Height (mm)	Width/Height	3 rods (RMS-e)	6 rods (RMS-e)	9 rods (RMS-e)
Leksell-G	120	120	1	0.711 (+/- 0.168)	0.591 (+/- 0.132)	0.579 (+/- 0.130)
BRW	140	189	0.741	0.581 (+/- 0.031)	0.533 (+/- 0.028)	0.525 (+/- 0.027)
Micromar	140	140	1	0.723 (+/- 0.159)	0.596 (+/- 0.125)	0.584 (+/- 0.123)
Fi.Me.	160	160	1	0.790 (+/- 0.169)	0.639 (+/- 0.131)	0.627 (+/- 0.129)

Localization areas

Table 1 demonstrates that the BRW LF is the most accurate and stable three N-localizer system among the four systems analyzed. A prior study on the accuracy of the BRW LF was also reported by Grunert in 1999 [7]. One explanation for this finding is that the overall volume that the N-localizer system encloses is larger, not only in the vertical dimension but also within each axial CT image. Enclosing more pixels within the image leads to more pixels per millimeter, minimizing errors via greater precision. Another important finding is the standard deviations of the targeting errors in each system. 

**Figure 2:**
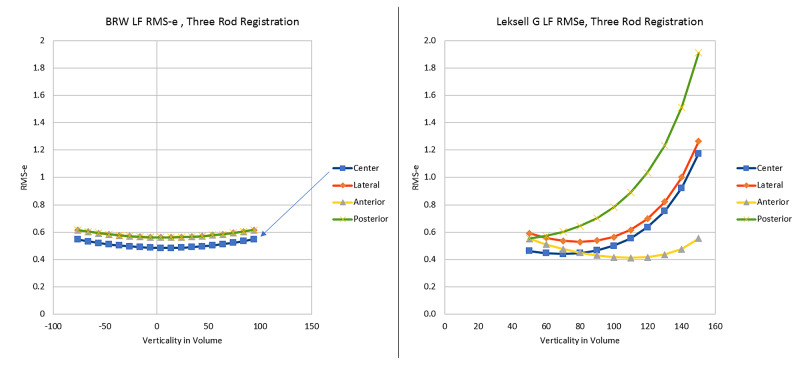
Comparative Monte Carlo simulations of Root Mean Square errors (RMS-e) over the volume of stereotactic space in the Brown-Roberts-Wells (BRW) localizer frame (LF) (left) and the Leksell G (LG) LF (right). The BRW LF is more stable over the entire volume of the stereotactic space. The LG LF is more accurate towards the cranial aspect of the stereotactic space, and less accurate especially at the inferior (higher numerical verticality) and posterior aspects. BRW, Radionics CRW Stereotactic System, Integra LifeSciences Corporation, Plainsboro, New Jersey Leksell G, Leksell Stereotactic Frame, Elekta, Stockholm, Sweden

To better understand where the RMS errors are occurring, we fix points 50 mm in each direction along the center, lateral, anterior, and posterior aspects of the entire volume. The results of MC simulations at these points are plotted for both the BRW LF and LG LF (Figure 2) and demonstrate that the BRW LF is relatively stable throughout the 50 mm space, but the LG LF has variable accuracy depending on the target location. The highest errors for LG LF are located at the inferior and posterior regions.

**Figure 3:**
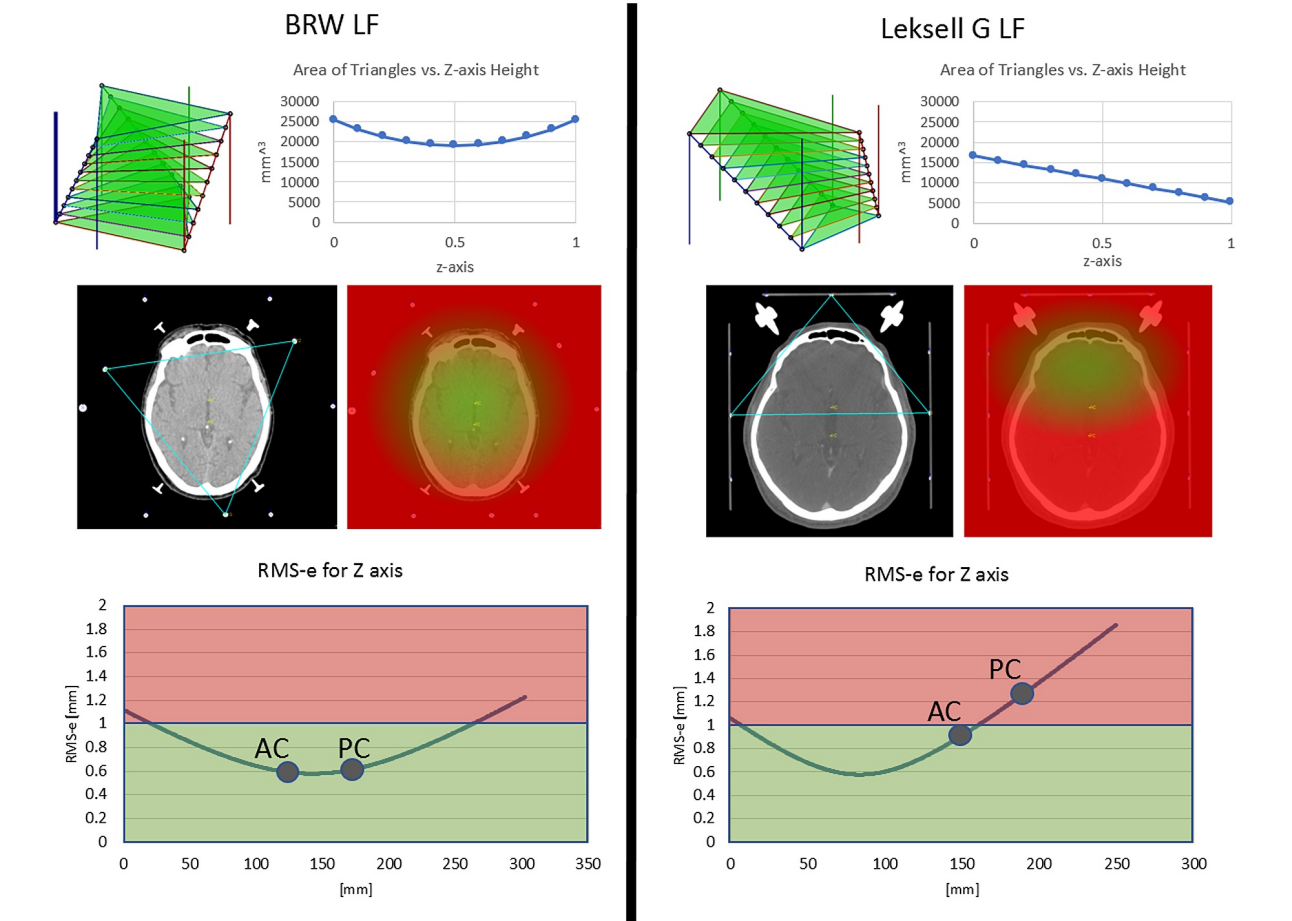
Depiction of the triangles formed by the fiducial points of the Brown-Roberts-Wells (BRW) localizer frame (LF) and Leksell G (LG) LF as well as the areas of the triangles over the normal to the z-axis (top images). For the BRW LF, the triangle’s area is nearly independent of the height of the axial computed tomography (CT) image. For the LG LF, the area varies according to the height of the CT image and is smaller for CT images that lie inferiorly. A smaller area correlates with a larger Root Mean Square error (RMS-e). Middle row of images are axial CT images showing the triangle formed by the diagonal rods and associated heat maps generated in MNPS that depicts an RMS-e for Z < (2*PixelSize) via green-shaded pixels computed using a barycentric representation of a linear property over the triangle and equation 36 in the Appendix. Note that the extent of the green heat map is much smaller for the LG LF than for the BRW LF. The pixel sizes for axial CT heat map images are 0.818 mm for BRW LF (bottom left) and LG LF (bottom right) using a 916x916 screen size matrix. Finally, RMS-e using Monte Carlo (MC) simulations for the BRW and LG LF are presented using three rods for the axial CT image (bottom row). In addition, the anterior commissure (AC) and posterior commissure (PC) are noted on the axial CT images and their associated locations are placed on the RMS-e curves. BRW, Radionics CRW Stereotactic System, Integra LifeSciences Corporation, Plainsboro, New Jersey Leksell G, Leksell Stereotactic Frame, Elekta, Stockholm, Sweden Computed Tomography (CT) MNPS - Mevis Stereotactic Planning System Area of Triangles - Courtesy of Ross Anderson, PhD

The apparent differences as depicted in Figure 2 between the BRW LF and LG LF may be related to an area of a triangle formed by the three-diagonal fiducials for each image plane. The idea of keeping the target point inside of this triangle was first proposed by Perry, et al. [8] and subsequently tested by Grunert, et al. [7] and Brown [3]. For the BRW LF, the area of such a triangle is fairly constant along the height (z) but for the LG LF, the area of the triangle varies significantly depending on the height (Figure 3). The largest predicted RMS-e for the LG LF occurs when a target lies posteriorly and inferiorly because for an inferior CT image, the triangle lies anteriorly and has a smaller area. Under these conditions, the target point has a higher probability of lying outside of the triangle. Heat maps can be generated using barycentric interpolation computed via Equation 36 in the Appendix, which are observed in Figure 3 for the BRW LF and LG LF [9-10]. For a more detailed analysis of these presumed errors related to targeting and the area of the triangle, please see the Appendix where two proofs demonstrate similar outcomes and introduce the term "stereotactic triangle". 

Given the above analysis of the areas of a triangle formed by the three diagonal rods, a simple modification to the two lateral N-localizer plates of the LG LF appears to be desirable. Specifically, the diagonal rods of these two N-localizer plates descend from posterior to anterior parallel to each other, as depicted in Figure 1. If instead, the right lateral N-localizer were inverted such that its diagonal rod descended from anterior to posterior, then all three diagonal rods would descend in a clockwise manner, similar to how the three diagonal rods of the BRW LF ascend in a clockwise manner. This modification of the LG LF would render the triangle area less sensitive to the height of the CT image, as is the case for the BRW LF. To explore the advantage of this inverted or antiparallel configuration, we evaluated a rectilinear 140x140 mm N-localizer system that comprises three N-localizers arranged in either standard (parallel) or inverted (antiparallel) configuration. The RMS-e for an overdetermined system of equations that exploits nine rods is 0.584 (+/- 0.123) mm for the standard configuration and 0.488 (+/- 0.093) mm for the inverted configuration. 

**Figure 4:**
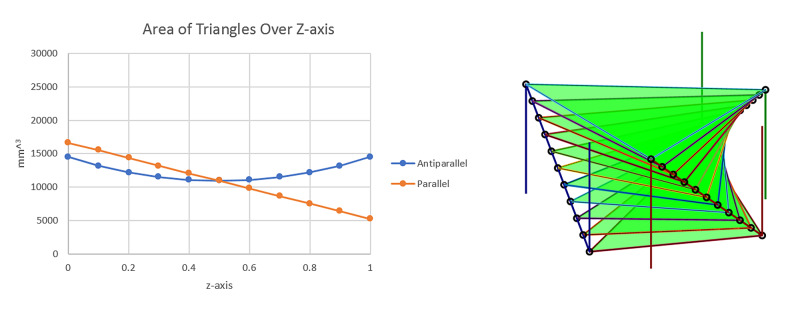
Antiparallel design and the effect on the area of triangles over the z-axis. Monte Carlo (MC) simulation for Root Mean Square error (RMS-e) suggested in the technical report that flipping one of the left or right panels of the Leksell G Localizer Frame (LF) would reduce targeting errors. For this reason, the areas of the triangles were analyzed for the parallel and antiparallel (inverted) N-localizer arrangements. Indeed, the antiparallel system insured uniformity of the area normal to the vertical axis (z) (left graph). Visually, it is observed that the triangles have more uniform areas (right image). Leksell G, Leksell Stereotactic Frame, Elekta, Stockholm, Sweden Courtesy of Ross Anderson, PhD

The effect of the antiparallel design of the three N-Localizer system on the area of the stereotactic triangle was next analyzed. The parallel N-localizer system, such as with the Leksell G (LG) Localizer Frame (LF) (Leksell G, Leksell Stereotactic Frame, Elekta, Stockholm, Sweden) exhibits a steady decline in the areas of triangles normal to the vertical axis (z) as presented in Figure 3. Indeed, the antiparallel arrangement creates more uniformity of the areas of triangles normal to the vertical axis (Figure 4).

Width versus height geometry of N-localizer systems

Monte Carlo analysis for RMS-e of the target can be performed using various sizes of N-localizer systems. For the BRW LF, we varied the vertical height subtended by the three N-localizers from 189 mm to 120 mm and used a nine-rod overdetermined system of equations to understand how height may affect the RMS-e. As expected, the average RMS-e improved from 0.51 (+/- 0.013) mm to 0.37 (+/- 0.008) mm as the height decreased (Table 2). 

**Table 2:** Monte Carlo (MC) simulations to determine average Root Mean Square Errors (RMS-e) were computed across the entire volume enclosed by the N-localizer system for various heights subtended by the Brown-Roberts-Wells (BRW) localizer frame (LF). RMS-e decreases with increasing the width/height ratio. The BRW LF (standard) is the commercial version, whereas the other versions are modified in only the vertical dimension. Millimeter (mm) Root Mean Square Error (RMS-e) BRW, Radionics CRW Stereotactic System, Integra LifeSciences Corporation, Plainsboro, New Jersey

Localizer	Width (mm)	Height (mm)	Width/Height	Average RMS-e Whole space (9 rods)
BRW LF (standard)	140	189	0.741	0.512 (+/- 0.014)
BRW LF (modified)	140	160	0.875	0.451 (+/- 0.011)
BRW LF (modified)	140	140	1	0.410 (+/- 0.010)
BRW LF (modified)	140	120	1.167	0.372 (+/- 0.008)

Conceptually, this effect can be explained by considering the interpolation along the N-localizer’s diagonal rod [4]. The steeper the diagonal rod, the greater the extent to which random noise will affect the interpolation in an interpolant. Therefore, the greater the width/height ratio of the N-localizer, the lower the RMS-e within that space. However, it should be noted that a considerable degree of height is necessary for localization throughout the entire stereotactic surgical space. Therefore, a balance between the practical limits to optimizing the width/height ratio and the encompassing space need to be considered. 

N-localizer systems with four N-localizers

An overdetermined system of equations minimizes errors in computing the transformation matrix (${ m }$). Previously, this overdetermined system had an influence on the x- and y-axes. However, the addition of another N-localizer adds more data for the x-axis, the y-axis, and, more importantly, the z-axis. Hence, incorporation of a fourth N-localizer to an N-localizer system that comprises three N-localizers may afford further improvement in RMS-e [3]. To explore this possibility, we used MC to analyze a 140x140 mm (WxH) rectilinear N-localizer system arranged in a standard configuration without inverting a diagonal rod. An incremental reduction in error is observed which is promoted by the incorporation of an overdetermined system of equations, here starting with four rods but reaching a total of twelve rod positions (Table 3). These errors may be compared to the error shown in Table 1 for the Micromar (Micromar Ind. Com. Ltda, Diadema, Sao Paulo, Brazil) (140x140 mm) three N-localizer system. Using maximum data for the overdetermined systems, the RMS-e for the three N-localizer Micromar and the four N-localizer systems that have comparative geometry are 0.58 (+/- 0.12) and 0.36 (+/- 0.018), respectively.

**Table 3:** Four N-Localizer system is analyzed using Monte Carlo (MC) simulations of Root Mean Square Errors (RMS-e) of different target locations using four, eight, and twelve rods. Using an overdetermined system of equations improves the RMS-e from four equations to twelve. One also observes good stability throughout the stereotactic volume given the low standard deviation. These errors may be compared to the error shown in Table 1 for the Micromar (Micromar Ind. Com. Ltda, Diadema, Sao Paulo, Brazil) (140x140 mm) three N-localizer system. Using maximum data for the overdetermined systems, the RMS-e for the three N-localizer Micromar and the four N-localizer systems that have comparative geometry are 0.58 (+/- 0.12) and 0.36 (+/- 0.018), respectively. Millimeter (mm) Micromar Ind. Com. Ltda, Diadema, Sao Paulo, Brazil

4 N-Localizer System			
140x140 mm	4 Rods (RMS-e)	8 Rods (RMS-e)	12 Rods (RMS-e)
Center	0.379	0.337	0.328
Lateral 50 mm	0.427	0.378	0.368
Frontal 50 mm	0.427	0.378	0.368
Posterior 50 mm	0.427	0.378	0.368
Average	0.415	0.368	0.358
Standard Deviation	0.021	0.018	0.018

## Discussion

The N-localizer is a critical component of most frame-based stereotactic neurosurgical procedures, which are often used for precise targeting such as for deep brain stimulation (DBS), brain biopsies, invasive electroencephalography (iEEG), and laser interstitial thermal therapy (LITT). The N-localizer allows every axial slice in the localizer imaging space to be localized precisely, which can then be used during the neurosurgical procedure. Despite the important use of the N-localizer, there are no focused publications that analyze various optimizations of the N-localizer that can be implemented. Herein, we report the first such approach to improve the technology. 

Since every image will have some associated noise that relatively uniform, using a system of overdetermined equations minimizes some of the errors during the stereotactic localization step. Introduction of an error during this step, therefore, may propagate into the actual surgical procedure, which may also have its own errors related to image fusion, imprecision from manually setting frame coordinates, and displacements related to imperfect instruments and deflections along a trajectory. Therefore, these targeting errors can be additive. Further, in comparing target accuracy for the CRW system versus the Leksell G (LG), average errors reported are 1.94 (+/- 0.41) versus 2.35 (+/- 1.03), respectively [11]. The larger average error for the LG relative to the CRW correlates well with the larger RMS-e predicted via MC for the LG Localizer Frame (LF) relative to the BRW LF (see Figure 1). While there are several factors and different methods that may affect these reported results, optimizing precision during the localization step may improve targeting for the neurosurgical procedure. In addition, techniques for improving Stereotactic Intraoperative Localization (StIL) and navigating coordinate systems have been previously reported [12-14]. 

There are several forms of optimization that play a role in error minimization. One such example is the novel utilization of all the vertical rod positions for their associated x- (LAT) and y- (AP) axes. As such, an overdetermined set of equations significantly reduces final computation errors for a target. This method is particularly attractive as it can be immediately performed on all N-localizer systems. 

Other improvements in localization include optimization of the geometry of the N-localizer. The area of a triangle formed by three diagonal rods relative to a target can also play a role in errors. Targeting within this stereotactic triangle may be key to minimizing the errors. This observation leads to relatively simple improvements such as inverting opposite diagonal rods (antiparallel) and considering larger overall structures. There will be CT/MRI limitations related to size and perhaps inhomogeneity further away from the center of the bore, which would require further study. Further, the slope of the diagonal bar can play a role in errors as is exemplified by observing the width versus height of the N-localizer system. 

**Figure 5:**
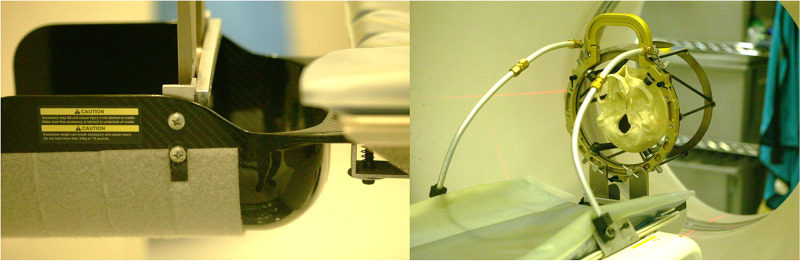
Computed Tomography (CT) Scanner attachments. The left image is a head-ring fixture which holds the head-ring of a patient during a CT scan acquisition. The right image shows the Brown-Roberts-Wells (BRW) Localizer Frame (LF) with the Cosman-Roberts-Wells (CRW) system being supported by the headring fixture and restrained with brackets. This type of arrangement reduces noise during image acquisition and allows the posterior N-Localizer to be imaged well. Courtesy of Eric Sabelman PhD

While many systems primarily utilize three N-localizer registration, higher precision appears to be gained by utilizing a four N-localizer system [3]. For square- or rectangular-shaped three N-localizer systems, generally, the plates used are the lateral and anterior ones to avoid the posterior plate, which may be distorted during a CT or MRI study, or have materials that impair proper rod localization. Therefore, routine use of systems to support/suspend the stereotactic frames and suppress movement artifact optimize imaging conditions. Care during image acquisition cannot be overemphasized (Figure 5). 

## Conclusions

Monte Carlo simulations of Root Mean Square error (RMS-e) is a useful technique in understanding targeting while using N-localizer systems in stereotactic neurosurgery. The authors have described many novel techniques that could lead to immediate improvements in the localization step. Some of these improvements include mathematical techniques and others are geometrical alterations of the systems. Utilizing all the fiducials of the N-localizer system and keeping the target within a stereotactic triangle may have clinical importance. Further investigation is needed to confirm these findings.

## Figures and Tables

**Figure 6:**
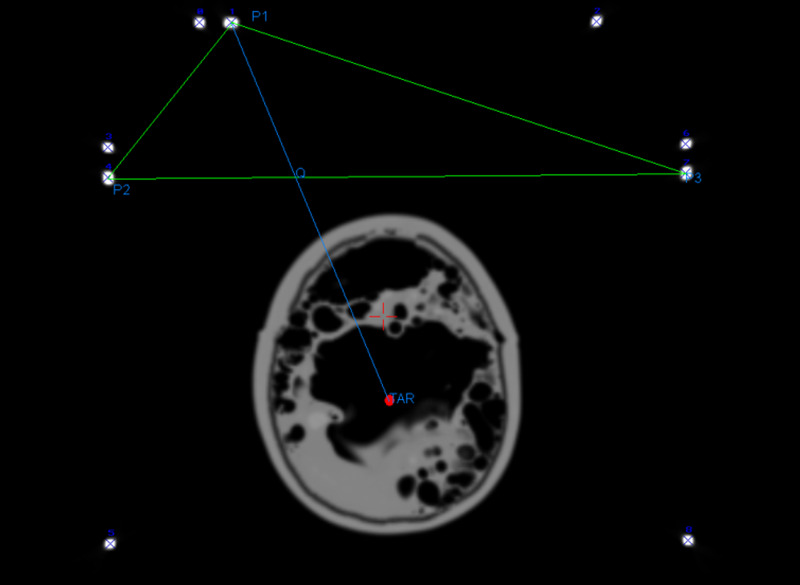
An axial Computed Tomography (CT) slice of a phantom with a 3 N-localizer system. The diagonal bars form three points ($P_{1}, P_{2}, P_{3}$). A triangle is formed by these three points. A target (TAR, $T$) within the phantom is placed, but this is outside the triangular area. There also exists a point ($Q$) which is the intersection of the line formed by $P_{1}$ - $T$ and $P_{2}$ - $P_{3}$.

**Figure 7:**
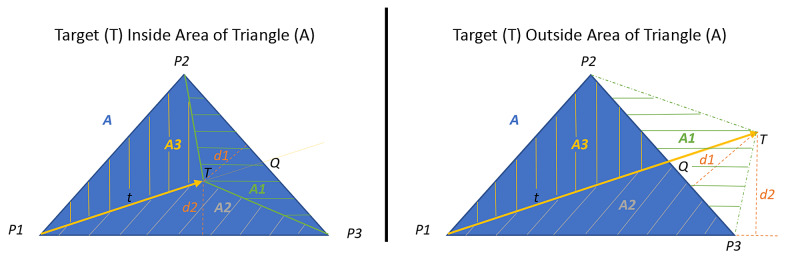
Area $A$ of Triangle define by points $P_{1}, P_{2}, P_{3}$ are related to a target $T$. This target $T$ may reside inside or outside of the area $A$. A vector can be formed from point $P_{1}$ to $T$; this vector intersects at point $Q$ on the line formed by $P_{2}$ and $P_{3}$. These lines and vectors form different area triangles $A_{1}, A_{2}, A_{3}$. The derivation of the proofs relates this geometry to define a function to describe potential errors.

**Figure 8:**
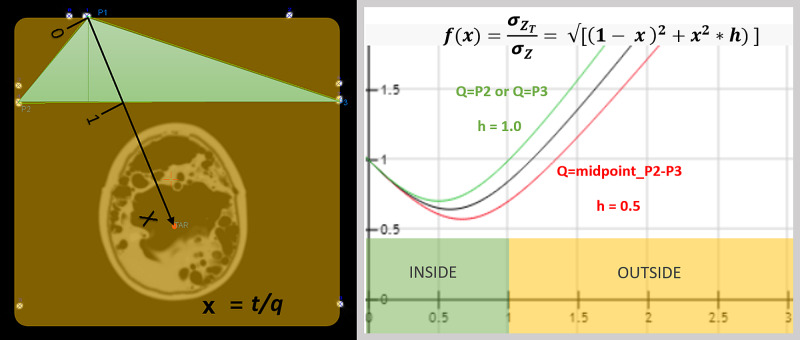
The function $f(x)$ from equation 36, which demonstrates potential errors was derived using Barycentric coordinates. Left image shows an axial computed tomography (CT) image whereby the three diagonal rods form a triangle. The right image shows the function as it relates to the target position. One can observe that the function minimizes at the center of the triangle, which is the valley seen between $x$ of 0.5 to 1. When the target extends beyond $Q$ outside the area of the triangle, the function increases. Therefore, the propagation of error is lower than 1 inside the triangle and increases outside it, getting larger the farther from the triangle the target is. From this observation, it is concluded that the probabilities that the target is within a triangle should be maximized. Or equivalently the area of the triangle should be maximized.

**Figure 9:**
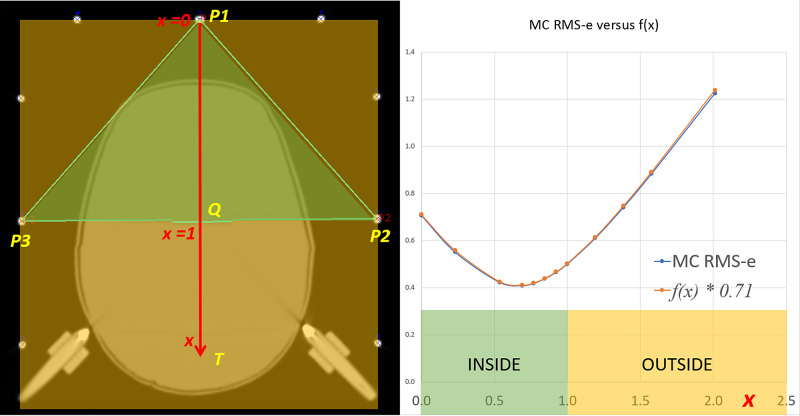
The function $f(x)$ in equation 60 may be used to describe potential errors. Left image shows an axial computed tomography (CT) image whereby the three diagonal rods form a triangle. The right image shows the function as it relates to the target position. One can observe that the function minimizes at the center of the triangle. When the target extends beyond $Q$ outside the area of the triangle, the function increases. Therefore, the propagation of error is lower than 1 inside the triangle and increases outside it, getting larger the farther from the triangle the target is. From this observation, it is concluded that the probabilities that the target is within a triangle should be maximized. Or equivalently the area of the triangle should be maximized. Note that the analytical graph overlaps well with the Monte Carlo (MC) simulation of Root Mean Square error (RMS-e) for this axial CT slice.

## Appendices

The Stereotactic triangle

As described in the technical report, the 3 diagonal N-localizer fiducials encode three 3D positions in addition to three 2D positions. Unlike the vertical bars, these diagonal bars are the only locations in 3D space that provide a value for the z-axis (vertical, z).  Therefore, study of errors related to the z is the focus of the two proofs, which support the evidence computed via Monte Carlo simulations provided in the technical report. 

Let us consider an axial computed tomography (CT) slice as a Euclidean plane containing 3 N-localizer fiducials.  From those 3 N-localizer fiducials, we have 3 points ($P_{1}, P_{2}, P_{3}$) at the diagonals.  From these data, a target point (TAR, $T$) will need to be computed. Computing the x (lateral, LAT)  and y (anteroposterior, AP) coordinates of the target will not be discussed here, because there are better methods available, as discussed in the technical report. For each diagonal point ($P_{1}, P_{2}, P_{3}$), their associated values for z carry random errors from noise.  The three points ($P_{1}, P_{2}, P_{3}$) form a triangle and the TAR in this case is presumed to lie outside of the triangle (Figure 6).  $T$ has an unknown z value.  Taking the diagonal rod farthest away from the target point ($P_{1}$), $Q$ is the intersection point between the line $P_{1}$ - $T$ and the line $P_{2}$ - $P_{3}$. The stereotactic target $T$ may be inside or outside of the triangle area (Figure 6).  We introduce the term, the "stereotactic triangle", to refer to this triangle for 3 N-localizer systems. We stipulate, therefore, that to ensure accurate targeting, a target should be within the area of this triangle and that errors of larger and larger magnitudes may arise as the target moves farther and farther outside of the triangle. 

For the two subsequent proofs, we define a general triangle using 3 points $P_{1}, P_{2}, P_{3}$ to develop a function using partial derivatives which will describe the error [15, 16]. We also define a target $T$ and a point $Q$ that intersects two lines formed by  $P_{1}, T$ and  $P_{2}, P_{3}$.  Next, let us define areas $A_{1}, A_{2}, A_{3}, A$ and vectors $t, q$ related to the triangle (Figure 7). 

Proof 1: General interpretation of the relation between z error and a triangle area using Barycentric Coordinates and partial differential equations

We next setup two vectors from $T$ to $P_{1}$ (equation 9) and $Q$ to $P_{1}$ (equation 10). The variable $x$ now relates the fractional distance of $t$ divided by $q$ (equation 11). The symbol := in the subsequent equations implies "equal by definition". 

$$\vec{TP_{1}} = T - P_{1}  = \vec{v}*t,  \vert \vec{v}  \vert = 1 \tag{9}$$

$$\vec{QP_{1}} = Q - P_{1}  = \vec{v}*q,  \vert \vec{v}  \vert = 1 \tag{10}$$

$$x := t/q \tag{11}$$

It can then be determined that there are subsections of the triangle that can be defined in fractions as $W_{2}$, $W_{3}$, $W_{1}$ (equations 12-14). We can then compute the target position $Z_{T}$ (equation 15).  It then follows that the areas and the fractional areas can be rearranged and combined to form equations 16-25. 

$$W_{2} := A_{2}/A \tag{12}$$

$$W_{3} := A_{3}/A \tag{13}$$

$$W_{1} := 1 - W_{2} - W_{3} \tag{14}$$

$$Z_{T} = W_{1}*Z_{1} + W_{2}*Z_{2} +W_{3}*Z_{3} \tag{15}$$

$$A_{2} = \vert     \vec{TP_{1}}    \times\vec{P_{1}P_{3}  }                  \vert /2 \tag{16}$$

$$A_{2} =  \frac{t}{2} * \vec{v} \times \vec{P_{1}P_{3}}  \tag{17}$$

$$A_{3} = \vert     \vec{TP_{1}}    \times\vec{P_{1}P_{2}  }                  \vert /2 \tag{18}$$

$$A_{3} =  \frac{t}{2} * \vec{v} \times \vec{P_{1}P_{2}}  \tag{19}$$

$$A =      \vert \vec{QP_{1}} \times   \vec{P_{1}P_{2}}       \vert /2   +          \vert    \vec{QP_{1}} \times   \vec{P_{1}P_{3}}     \vert /2           \tag{20}$$

$$A =      \frac{q}{2}   *   ( \vert  \vec{v} \times   \vec{P_{1}P_{2}}   \vert     +  \vert    \vec{v} \times   \vec{P_{1}P_{3}}      \vert          )     \tag{21}$$

$$W_{2} = \frac{A_{2}}{A}  =    \frac{t}{q} * (  \frac{  \vert  \vec{v} \times   \vec{P_{1}P_{2}}   \vert}{ \vert  \vec{v} \times   \vec{P_{1}P_{2}}   \vert     +  \vert    \vec{v} \times   \vec{P_{1}P_{3}}      \vert }        )     =      \frac{t}{q} * \frac{1}{1 + \frac{\vert    \vec{v} \times   \vec{P_{1}P_{3}}      \vert}{\vert    \vec{v} \times   \vec{P_{1}P_{2}}      \vert}  }    =   x * \frac{1}{1 + \frac{d_{2}}{d_{3}}}   = x*C_{2}   \tag{22}$$

$$W_{3} = \frac{A_{3}}{A}  =    \frac{t}{q} * (  \frac{  \vert  \vec{v} \times   \vec{P_{1}P_{3}}   \vert}{ \vert  \vec{v} \times   \vec{P_{1}P_{2}}   \vert     +  \vert    \vec{v} \times   \vec{P_{1}P_{3}}      \vert }        )     =      \frac{t}{q} * \frac{1}{1 + \frac{\vert    \vec{v} \times   \vec{P_{1}P_{2}}      \vert}{\vert    \vec{v} \times   \vec{P_{1}P_{3}}      \vert}  }    =   x * \frac{1}{1 + \frac{d_{3}}{d_{2}}}   = x*C_{3}   \tag{23}$$

$$(C_{2} + C_{3} ) = 1  \tag{24}$$

$$W_{1} = 1- x (C_{2} + C_{3} ) = 1 -x   \tag{25}$$

We now choose to parameterize  $Z_{T}$ further to develop a function  $f(x)$. Taking equation 15, we now introduce equations 22, 23, and 25 into a combined form (equation 26). We now take partial differentials (equations 27-29). Rearranging equations 27-29, taking their squares, we can then combine them to form equation 30.  We make the assumption that the noise in the function is homogenous (equation 31). Finally, defining a parameter $h$ with boundaries (equation 33-34), then we arrive at equation 35.  The function which may describe an error is then defined in equation 36. 

$$Z_{T} =  W_{1}*Z_{1} +  W_{2}*Z_{2} +W_{3}*Z_{3}  = (1-x)*Z_{1}  + x*C_{2}*Z_{2}    + x*C_{3}*Z_{3}             \tag{26}$$

$$\frac{ dZ_{T}}{dZ_{1}} = (1- x)            \tag{27}$$

$$\frac{ dZ_{T}}{dZ_{2}} = x*C_{2}            \tag{28}$$

$$\frac{ dZ_{T}}{dZ_{3}} = x*C_{3}            \tag{29}$$

$$\sigma_{Z_{T}}^2   = (1-x)^2*\sigma_{Z_{1}}^2  + x^2*C_{2}^2*\sigma_{Z_{2}}^2  + x^2*C_{3}^2*\sigma_{Z_{3}}^2     \tag{30}$$

$$\sigma_{Z}^2  := \sigma_{Z_{1}}^2 \approx \sigma_{Z_{2}}^2 \approx \sigma_{Z_{3}}^2   \space  (homogenous \space noise)   \tag{31}$$

$$\sigma_{Z_{T}}^2  = (  (1-x)^2 + x^2*( C_{2}^2 + C_{3}^2 )      ) *\sigma_{Z}^2      \tag{32}$$

$$h  :=    ( \frac{1}{1+  \frac{d_{3}}{d_{2}}   })^2   +  (\frac{1}{1+  \frac{d_{2}}{d_{3}}   })^2 \tag{33}$$

$$0.5      \leqslant      h  \leqslant 1  \tag{34}$$

$$\sigma_{Z_{T}}  =  \sqrt{  ( (1-x  )^2 + x^2*h   ) }*\sigma_{Z}               \tag{35}$$

$$f(x) =  \sigma_{Z_{T}}/\sigma_{Z}  =  \sqrt{  ( (1-x  )^2 + x^2*h   )          }      \tag{36}$$

The function from equation 36 demonstrates that error is in fact minimized in the center of the triangle (Figure 8). 

Proof 2: Geometric interpretation of the relation between Z error (Vertical) and the size/position of the triangle subtended by the diagonal rods using Partial Differentials. 

First, let us define the vectors $t$ and $q$, (equation 37 and 38) and their ratio $x$ (equation 39).  $Z_{Q}$, the z value at point $Q$, can then be computed (Equation 40).  We now define a value $C$ which defines the fractional distance of $Q$ relative to $P_{2}$ and $P_{3}$ (equations 41, 42). From this, we can redefine the z value at point $Q$ (equation 43) and at the Target ($T$) (equation 44).

$$t := \vert\vec{P_{T}P_{1}}\vert \tag{37}$$

$$q := \vert\vec{P_{Q}P_{1}}\vert \tag{38}$$

$$x := t/q \tag{39}$$

$$Z_{Q} = Z_{2} + (Z_{3}-Z_{2})*(\vert\vec{P_{Q}P_{2}}\vert / \vert\vec{P_{Q}P_{2}}\vert)  \tag{40}$$

$$C := \vert\vec{P_{Q}P_{2}}\vert / \vert\vec{P_{3}P_{2}}\vert \tag{41}$$

$$0 \leqslant  C \leqslant  1 \tag{42}$$

$$Z_{Q} = Z_{2} + (Z_{3}-Z_{2})*C  \tag{43}$$

$$Z_{T} = Z_{1} + (t/q) * (Z_{Q} - Z_{1}) \tag{44}$$

However, we are interested in errors related to $Z_{T}$. We now utilize differential equations for further analysis.  From equation 44, $dZ_{T}/dZ_{1}$ can be computed as can $dZ_{T}/dZ_{Q}$ (equation 45, 46).  It then follows by rearranging and combining equations 45 and 46 that equations 47 and 48 can be derived. We simplify the terms by using the previously defined $C$ (equations 49, 50) and add a parameter $x$ (equations 51, 52). 

$$\frac{ dZ_{T}}{dZ_{1}} = (1- x)  = 1.0 - x  \tag{45}$$

$$\frac{ dZ_{T}}{dZ_{Q}} = x\tag{46}$$

$$\frac{ dZ_{T}}{dZ_{2}} = \frac{ dZ_{T}}{dZ_{Q}}*\frac{ dZ_{Q}}{dZ_{2}} = x*\frac{ dZ_{Q}}{dZ_{2}} \tag{47}$$

$$\frac{ dZ_{T}}{dZ_{3}} = \frac{ dZ_{T}}{dZ_{Q}}*\frac{ dZ_{Q}}{dZ_{3}} = x*\frac{ dZ_{Q}}{dZ_{3}} \tag{48}$$

$$\frac{ dZ_{Q}}{dZ_{2}} = 1-C \tag{49}$$

$$\frac{ dZ_{Q}}{dZ_{3}} = C \tag{50}$$

$$\frac{ dZ_{T}}{dZ_{2}} = x*(1-C) \tag{51}$$

$$\frac{ dZ_{T}}{dZ_{3}} = x*C \tag{52}$$

Using equations 45, 51, and 52, we rearrange the terms, take the square, and combine them to form the parameterized function sigma squared (equation 53).  We now make the assumption that the noise in this system is homogenous (equation 54) as in proof 1.  We can then simplify the terms (equation 55-57).  Introducing the value h, for simplicity and keeping the value equal to or between 0.5 and 1, we have equations 58 and 59.  Finally, a function that may describe errors is defined in equation 60.   

$$\sigma_{Z_{T}}^2   = \frac{ dZ_{T}}{dZ_{1}}^2*\sigma_{Z_{1}}^2  + \frac{ dZ_{T}}{dZ_{2}}^2*\sigma_{Z_{2}}^2  + \frac{ dZ_{Q}}{dZ_{3}}^2*\sigma_{Z_{3}}^2     \tag{53}$$

$$\sigma_{Z}^2 := \sigma_{Z_{1}}^2 \approx \sigma_{Z_{2}}^2 \approx \sigma_{Z_{3}}^2   \space  (homogenous \space noise)   \tag{54}$$

$$\sigma_{Z_{T}}^2   = ( \frac{ dZ_{T}}{dZ_{1}}^2*  + \frac{ dZ_{T}}{dZ_{2}}^2*  + \frac{ dZ_{Q}}{dZ_{3}}^2 )*\sigma_{Z}^2     \tag{55}$$

$$\sigma_{Z_{T}}^2   = ( (1- x)^2*  + x^2*(1-C)^2*  + x^2*C^2 )*\sigma_{Z}^2     \tag{56}$$

$$\sigma_{Z_{T}}^2   = ( (1- x)^2*  + x^2*h)*\sigma_{Z}^2     \tag{57}$$

$$h := (1-C)^2  + C^2 \tag{58}$$

$$0.5 \leqslant  h \leqslant  1 \tag{59}$$

$$f(x) = \sigma_{Z_{T}} / \sigma_{Z}   = \sqrt{ (1- x)^2+x^2*h }     \tag{60}$$

The function determined in equation 60 yields the same function (Figure 9) supported in proof 1 and with the Monte Carlo simulations (see Figure 3 of the technical report). These data offer further mathematical proof for potential errors incurred by targeting outside the stereotactic triangle. 
